# Monkeypox-Associated Genital Necrosis in an Immunocompromised Patient: Case Report and Literature Review of Genital Mpox Reconstruction

**DOI:** 10.1055/a-2856-5092

**Published:** 2026-07-07

**Authors:** Nae-Ho Lee, Beom Jin Lim, Jin Yong Shin, Yoon Kyu Chung, Si-Gyun Roh

**Affiliations:** 65377 Department of Plastic and Reconstructive Surgery, Research Inst. of Clinical Medicine of Jeonbuk National University-Biomedical Research Inst. of Jeonbuk National Univ. Hospital, Jeonju, Korea

**Keywords:** soft tissue injuries, wound healing, reconstructive-lower extremities, Monkeypox, HIV infections, plastic surgery procedures, penile diseases

## Abstract

Monkeypox (Mpox) is a viral infection that has shown increased global incidence, particularly affecting immunocompromised populations. We present a case of a 45-year-old human immunodeficiency virus (HIV)-positive man with progressive penile ulceration and necrosis. Mpox was confirmed via polymerase chain reaction testing of skin and oropharyngeal lesions. Despite antiviral therapy and antibiotics, the patient developed full-thickness necrosis of the penile shaft, scrotum, and suprapubic region. Surgical incision, drainage, and debridement were performed, followed by reconstruction using a pedicled anterolateral thigh flap. Secondary intention healing was selected for the penile shaft to preserve tissue elasticity and erectile function. The patient achieved complete wound healing within 3 months without complications. This case highlights a severe and rare manifestation of mpox in the setting of advanced HIV infection, emphasizing the importance of early diagnosis, multidisciplinary management, and tailored surgical planning to optimize both functional and aesthetic outcomes in immunocompromised patients.

## Introduction

Monkeypox (Mpox) is an emerging zoonotic disease caused by the mpox virus (MPXV). MPXV is a DNA virus classified within the genus *Orthopoxvirus* and produces a clinical illness resembling smallpox. Mpox is pathogenic in both animals and humans, but its primary host remains unidentified. The first recognized human case of mpox occurred in 1970 in the Republic of the Congo, Central Africa. By May 2022, more than 3,000 cases had been reported across over 50 countries, prompting the World Health Organization to declare an “evolving threat of moderate public health concern” on June 23, 2022. 1 2 3

MPXV transmission occurs primarily through large respiratory droplets and direct contact with skin lesions. Sexual transmission has also been identified in this outbreak, with many cases presenting with genital and perianal lesions. 3 4

Patients with human immunodeficiency virus (HIV) infection, especially those experiencing advanced immunosuppression, may present with more severe manifestations of mpox infection. These can include extensive skin involvement, delayed wound healing, secondary infections, and rare complications such as tissue necrosis. 5 6 Also, these can involve additional organ systems, leading to dysfunction in organs such as the liver, lungs, heart, brain, eyes, or gastrointestinal system. 7 While anogenital involvement is common, there is still limited clinical evidence regarding the progression to full-thickness genital necrosis that necessitates complex surgical reconstruction. Specifically, the optimal surgical timing and the selection of reconstructive methods, such as the use of regional flaps versus conservative management for erectile function preservation, remain poorly defined in this vulnerable population.

Here, we describe a case involving a 45-year-old male patient with mpox, who was HIV-positive and presented with necrosis of the penile skin. The clinical progression, lesion morphology, and histopathological findings in this case provide insight into an atypical and severe form of mpox infection. We aim to highlight the importance of expanding the clinical understanding of mpox, particularly among high-risk immunocompromised populations.

The patient provided written informed consent for the publication of this case report and any accompanying images. Institutional review board approval was obtained in accordance with the ethical standards of our institution.

## Case

A 45-year-old man presented to the hospital with progressive penile swelling and ulceration. He reported a history of unprotected receptive anal intercourse with a male partner approximately 1 month before the onset of symptoms. He initially visited a local clinic due to painful penile ulcers and swelling, and serologic testing revealed a new diagnosis of HIV. He was subsequently referred to our institution for further evaluation and management.

On genital examination, the patient had extensive swelling and ulceration of the penile shaft, glans, scrotum, and pubic region, with clear evidence of superimposed infection and necrosis ( Fig. 1 ). And physical examination revealed extensive penile and scrotal edema accompanied by necrosis, multiple vesicular and ulcerative skin lesions scattered across the entire body, and palpable bilateral inguinal lymphadenopathy ( Fig. 2 ). Pelvic computed tomography (CT) demonstrated an 8-mm fluid collection along the ventral aspect of the penile shaft, suggesting a penile abscess ( Fig. 3 ).

**Fig. 1:**
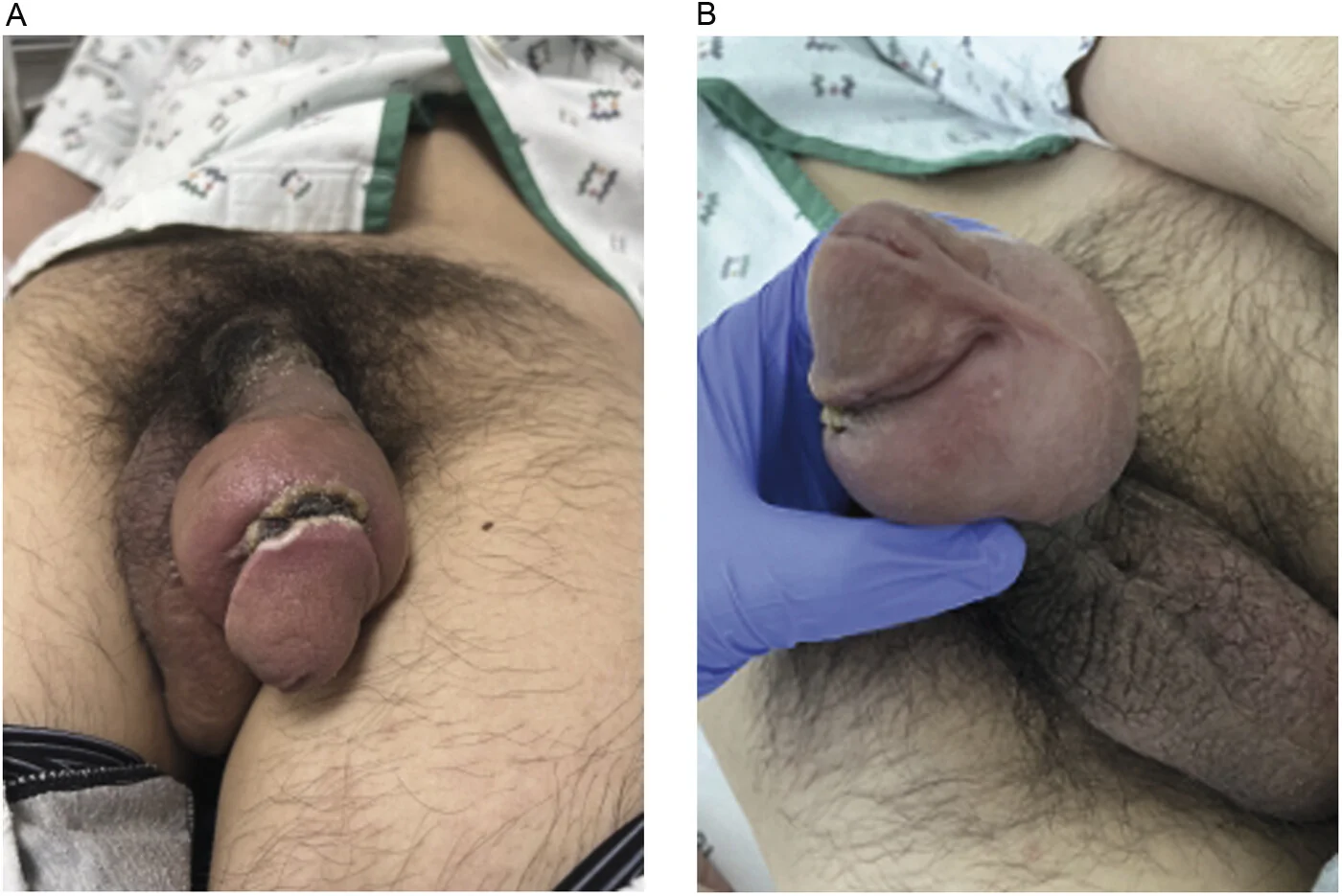
Extensive genital necrosis at presentation. Clinical photograph showing marked edema and ulceration involving the penile shaft, glans, scrotum, and pubic region, with areas of superimposed infection and necrosis.

**Fig. 2:**
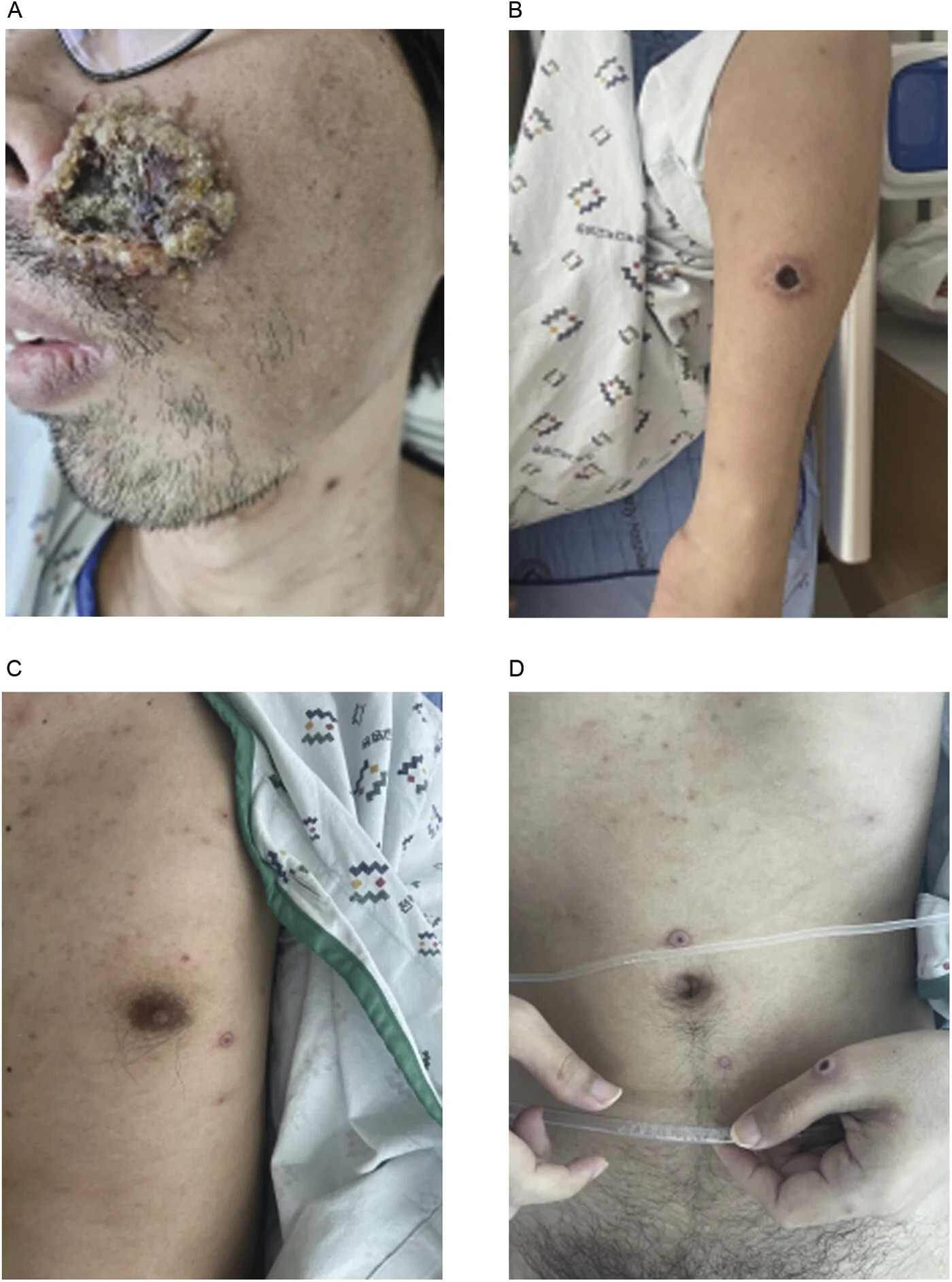
Disseminated vesiculoulcerative skin lesions associated with mpox. Multiple vesicular and ulcerative skin lesions observed in various body regions. ( **A** ) Crusted and ulcerative lesions on the face. **(B)** Erythematous vesiculoulcerative lesions on the forearm. ( **C** ) Multiple ulcerative lesions on the chest. ( **D** ) Discrete vesicular lesions on the abdomen.

**Fig. 3:**
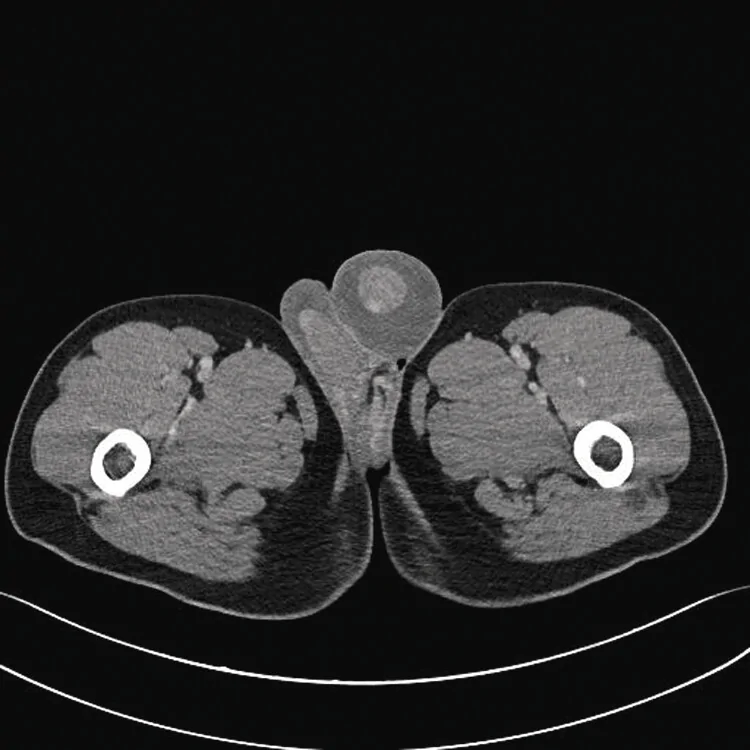
Pelvic CT of penile abscess. Axial pelvic CT showing an 8-mm ventral fluid collection along the penile shaft, consistent with penile abscess. CT, computed tomography.

The specimens collected from the oropharynx, plasma, and vesiculoulcerative skin lesions were sent to the regional Institute of Health and Environment Research, an organization affiliated with local government, which confirmed the mpox via polymerase chain reaction.

Upon admission, highly active antiretroviral therapy was initiated. The CD4+ T cell count at admission was 11 cells/µL, and HIV viral load was 5.9 log RNA copies/mL, consistent with advanced, untreated HIV infection. Highly active antiretroviral therapy (HAART) for HIV was started. One week later, intravenous tecovirimat was initiated for the treatment of mpox. Serologic testing for syphilis revealed a reactive Rapid Plasma Reagin titer of 1:2 and a strongly positive *Treponema pallidum* particle agglutination at 1:2560, consistent with prior or latent syphilis infection.

Then, surgical incision and drainage were performed promptly under general anesthesia. Culture of the pus revealed mixed bacterial flora, and empirical intravenous antibiotics were initiated. Daily antiseptic wound care was maintained, and mupirocin ointment was applied to the facial and peripheral lesions, which gradually improved over the subsequent 10 days.

In contrast, the penile and perineal lesions advanced to deep ulceration with full-thickness necrosis of the skin and subcutaneous tissue, despite antimicrobial therapy and local wound care. The necrotic region included the distal penile shaft, ventral scrotum, and lower pubic area. The boundaries of the wound became well-defined, prompting surgical debridement to remove all devitalized tissue and reduce the infectious burden ( Fig. 4 ).

**Fig. 4:**
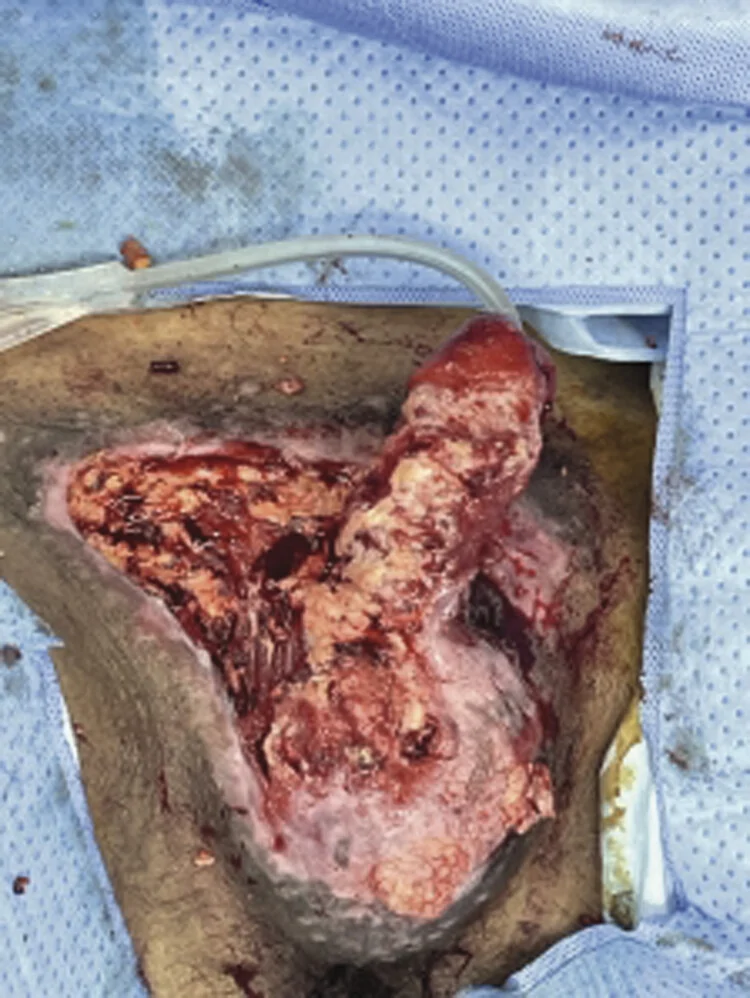
Wound after surgical debridement of necrotic genital tissue. Full-thickness necrosis involving the distal penile shaft, ventral scrotum, and lower pubic area after surgical excision of devitalized tissue.

Due to the extensive soft tissue defect and the requirement for durable coverage of the suprapubic region, a pedicled anterolateral thigh (ALT) flap was planned. Local options such as superficial circumflex iliac artery perforator (SCIP) or superficial external pudendal artery perforator (SEPA) flaps were considered; however, they were deemed unsuitable because of limited tissue volume, short pedicle length, and risk of partial necrosis in the setting of active infection. The ALT flap was chosen for its robust vascularity, reliable size, and ability to provide stable coverage in an immunocompromised host. Under general anesthesia, the flap was harvested from the left thigh, based on a perforator from the descending branch of the lateral circumflex femoral artery. Measuring 13 × 7 cm, the flap was elevated in the suprafascial plane. A subcutaneous tunnel was created from the left groin to the suprapubic region, and the flap was passed through without tension. The flap was then inset to cover the exposed suprapubic area, and the donor site was closed primarily with drainage. Areas of the penile shaft and scrotum with relatively well-vascularized wound beds were managed conservatively with regular dressings to allow for secondary intention healing ( Fig. 5 ). Skin grafting over the penile shaft was avoided, not because grafting is contraindicated, but to preserve elasticity and minimize the risk of secondary contracture, particularly in a circumcised patient with limited redundant skin. Although direct evidence of graft-related functional impairment is scarce, the penile shaft's gliding property is known to be essential for erection, and grafted skin can become relatively inelastic. Considering the patient's circumcised state, immunocompromised condition, and residual infection, secondary intention healing was deemed the most physiologic and safe option.

**Fig. 5:**
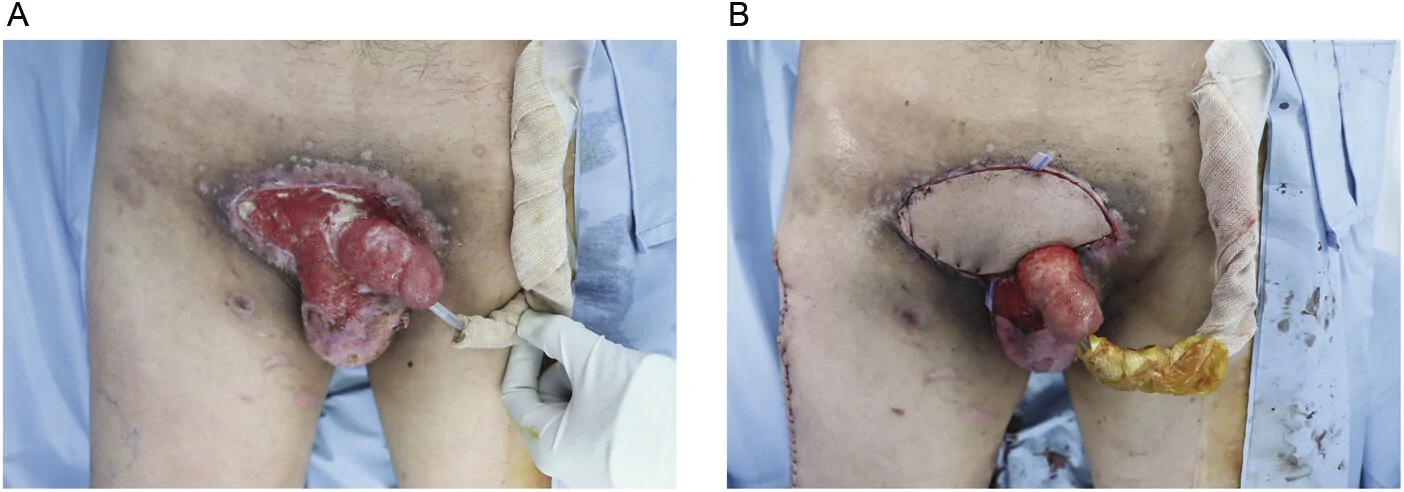
Reconstruction of suprapubic defect using a pedicled ALT flap. ( **A** ) Preoperative view showing the extensive soft tissue defect involving the suprapubic region. ( **B** ) Postoperative view after reconstruction with a pedicled ALT flap. The flap was tunneled subcutaneously to the defect site and inset. ALT, anterolateral thigh.

Postoperatively, the patient was monitored closely. The flap remained viable with no signs of ischemia, infection, or dehiscence. Daily dressing changes continued at both donor and recipient sites. Over 2 weeks, the wounds demonstrated progressive healing and good graft integration.

At the 3 months postoperatively, outpatient follow-up revealed complete wound healing with stable flap coverage and no complications ( Fig. 6 ). The patient's overall condition improved significantly, and he remained under follow-up in both the infectious disease and plastic surgery outpatient clinics. The patient maintained normal urination without difficulty. Assessment of sexual function was not feasible at the 3-month visit; however, long-term follow-up has been planned to evaluate erectile function and penile skin elasticity.

**Fig. 6:**
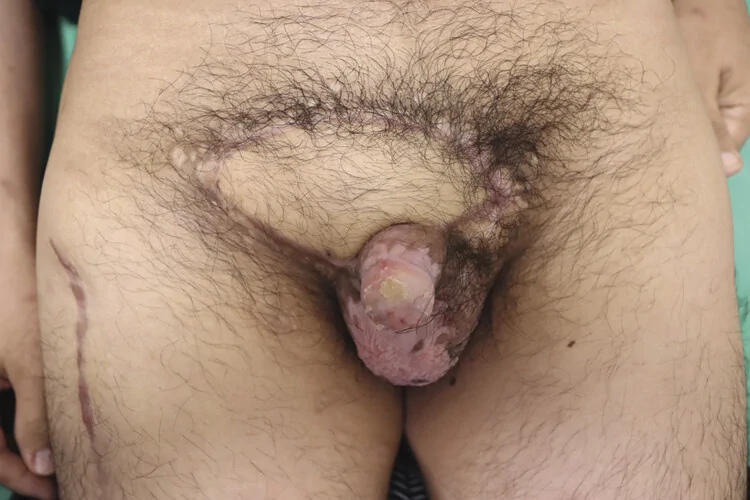
Follow-up view demonstrating stable flap and wound healing. Three months postoperative follow-up clinical photograph. The patient was followed up for 3 months without complications, demonstrating stable flap coverage and complete wound healing.

## Discussion

The recent global outbreak of mpox has greatly broadened our understanding of its clinical spectrum. Historically, mpox was largely confined to Central and West Africa, but the emergence of cases in over 50 countries—especially among sexually active populations—has revealed the diverse manifestations of this zoonotic infection. 3 This case, involving an HIV-positive patient who developed genital and perineal necrosis, highlights the severe consequences of mpox in immunocompromised individuals and provides valuable insight into its potential to cause rare and life-threatening complications.

In immunocompromised individuals, particularly those with untreated or advanced HIV infection, mpox often progresses in a more severe and protracted manner than in immunocompetent hosts. The compromised immune system fails to mount an adequate response to the virus, resulting in increased viral replication and more extensive tissue involvement. 8 9 In this case, the patient's profoundly low CD4+ T cell count (11 cells/µL) and high HIV viral load (5.9 log RNA copies/mL) likely contributed to the development of full-thickness skin necrosis and deep abscess formation—findings rarely seen in otherwise healthy individuals. This is consistent with an expanding body of literature that associates advanced HIV infection with atypical mpox presentations, including increased viral shedding, more widespread mucocutaneous involvement, and heightened susceptibility to secondary bacterial infections, as observed in this patient. 10 11 The severity in our patient can be attributed to the synergistic effect of advanced HIV and MPXV. Advanced HIV (CD4+ < 50 cells/µL) is known to impair cell-mediated immunity, which is crucial for controlling Orthopoxvirus replication. This leads not only to prolonged viral shedding but also to an exaggerated inflammatory response and secondary vascular compromise, which may manifest as necrotizing lesions similar to those seen in our case.

Reports of severe mpox in immunocompromised individuals are becoming increasingly common, suggesting that these patients may display unique clinical features such as more severe ulceration, delayed wound healing, and rare complications like necrotizing fasciitis or widespread soft tissue necrosis. 9 In the context of HIV, impaired immune defenses may delay viral clearance, further exacerbating disease progression. Several studies indicate that such patients are prone to prolonged infection with persistent lesions, which resolve more slowly than in immunocompetent hosts. 12 13 This case adds to the growing evidence that individuals with advanced HIV infection are at significantly increased risk for severe mpox manifestations, underscoring the importance of early diagnosis and aggressive management.

Genital lesions have become recognized as a frequent presentation of mpox during the 2022 outbreak, with many cases involving the genital and perianal regions. According to recent studies, anogenital lesions were documented in 73% of cases. 3 14 These lesions typically appear as shallow, self-limited ulcers that resolve with conservative management. 15 However, the present case demonstrates a rare and severe form of genital involvement, characterized by extensive necrosis and abscess formation affecting the penile and scrotal regions. Progression of mpox to full-thickness skin necrosis is exceedingly rare, particularly in the genital area. The presence of extensive necrosis and abscesses, in the absence of systemic symptoms such as fever, may challenge clinicians in distinguishing mpox from other serious conditions such as Fournier's gangrene, which also presents with necrotizing genital infections.

The co-occurrence of syphilis in this patient further complicated the clinical scenario. Syphilis, a well-established sexually transmitted infection (STI), is known to cause genital ulcers; however, the development of necrotic ulcers and abscesses in a patient with confirmed mpox renders the case, especially challenging in both diagnosis and management. This dual infection underscores the necessity of maintaining a high index of suspicion for other STIs in patients presenting with atypical genital ulcers, especially among HIV-positive individuals who are at increased risk for concurrent infections. 16 17

Although most cases of mpox resolve with supportive care and conservative wound management, surgical intervention became necessary in this patient due to the progression to deep tissue involvement. Early incision and drainage played a crucial role in controlling the local infection and preventing further complications such as systemic sepsis or gangrene. 18 As necrotic tissue became demarcated, surgical debridement was performed to remove all devitalized areas, optimizing the wound bed for reconstruction.

The selection of a pedicled ALT flap for reconstruction was based on the need for durable, well-vascularized coverage of the penile shaft and surrounding tissues. The ALT flap is a well-established, versatile reconstructive technique that provides both functional and aesthetic benefits. Its application in this case enabled optimal coverage of the defect while minimizing donor site morbidity and promoting rapid wound healing. 19 While skin grafts are simpler, they often undergo secondary contracture, which can significantly impair penile elasticity and erectile function, a critical concern in genital reconstruction. Regional flaps like the SCIP and SEPA flaps are alternative options. However, in the setting of active mpox and syphilis co-infection, the reliability of the vascular pedicle and the volume of healthy tissue available are paramount. Our choice of the pedicled ALT flap is supported by its robust vascularity and large skin paddle, which allowed for stable coverage even in a contaminated, immunocompromised environment. This careful, combined approach—aggressive surgical debridement followed by conservative management of viable tissue—helped promote healing and reduce the risk of complications at the donor site.

Management of mpox in immunocompromised patients presents several unique challenges. These individuals are at heightened risk for atypical disease courses, deep tissue necrosis, secondary bacterial infections, and delayed wound healing. 8 9 Moreover, comorbidities such as syphilis can further complicate diagnosis and treatment. Multidisciplinary care, involving infectious disease specialists and reconstructive surgeons, is essential to achieve optimal outcomes in these complex cases.

Additionally, this case illustrates the importance of timely diagnosis and prompt intervention. In mpox, early identification of lesions and initiation of appropriate therapy can reduce the risk of severe outcomes. 20 Clinicians must also recognize the potential for co-infection with other STIs in high-risk populations, including men who have sex with men (MSM) and individuals with HIV.

This case underscores the need for increased clinical vigilance regarding mpox, particularly during the ongoing global outbreak. As mpox continues to spread beyond endemic areas, clinicians must be prepared to identify and manage a wide range of clinical presentations—especially in high-risk populations such as those with HIV. This includes not only recognizing classic cutaneous lesions but also being alert to severe genital or systemic involvement that may necessitate surgical management.

A comprehensive literature review revealed that genital manifestations of mpox are relatively common, but necrosis requiring reconstruction is extremely rare. Cases of mpox in advanced HIV infection often demonstrate atypical severity, prolonged viral shedding, and delayed wound healing. Table 1 summarizes published reports of genital mpox with necrotic or ulcerative presentations, highlighting differences in comorbidity, management, and outcomes. 15 18 21 22 23 24 25 Our case adds to this body of evidence by documenting successful use of a pedicled ALT flap in combination with conservative penile management.

**Table 1:** Reported cases of genital mpox with necrotic or ulcerative presentation

Author (year)	Comorbidity/immune status	Lesion site	Management	Outcome
Miyazaki et al., 2024 (J Infect Chemother)	Chlamydia infection 1 mo prior; otherwise healthy MSM on PrEP	Perianal lesion; penile edema with black pigmentation at coronal sulcus	Conservative care and antiviral therapy (tecovirimat not specified)	Resolved without necrosis or functional sequelae
Umenzekwe et al., 2023 (Eur J Clin Med)	Immunocompetent; HIV, HBV, HCV negative	Solitary penile ulcer (3 × 2.5 cm) with satellite pustules and inguinal lymphadenopathy	Empirical antibiotics, mupirocin ointment, supportive therapy; no antiviral used	Complete resolution by day 14 (21 d after onset); stable on outpatient follow-up
Farley et al., 2023 (Urol Case Rep)	HIV positive; CD4 140 cells/µL	Penile shaft necrosis with edema	Debridement, wound-vac, scrotoplasty with A-cell, tecovirimat + antibiotics	Healed by 10 wk; good cosmetic result
Gawargeous et al., 2023 (Cureus)	Immunocompetent; HIV, HBV, HCV negative	Foreskin necrosis with penile ulcer and inguinal abscess	Urgent circumcision and debridement; IV antibiotics	Complete healing by 5 wk; PCR negative
Koh et al., 2023 (J Cutan Pathol)	Case 1: HIV positive (noncompliant > 1 y); syphilis positive Case 2: HIV negative, syphilis history	Case 1: Ulceronecrotic nodules and plaques (face, torso, extremities) Case 2: Two ulcerated papules on penile coronal sulcus	Tecovirimat (case 1); supportive care (case 2)	Case 1: Improved after 15 d of tecovirimat Case 2: Self-resolved by day 36
Abrefa Safo Y et al., 2025 (Cureus)	No known comorbidity	Vulva (labia majora/minora), perineum	Supportive care, broad-spectrum antibiotics	Resolved after isolation and conservative care
Alhalaseh et al., 2024 (Am J Dermatopathol)	HIV positive (poorly controlled)	Perianal, rectal ulcer, diffuse cutaneous lesions	Tecovirimat, IV antibiotics	Death (due to severe disease progression and complications)

HBV, hepatitis B virus; HCV, hepatitis C virus; HIV, human immunodeficiency virus; IV, intravenous; PrEP, preexposure prophylaxis; PCR, polymerase chain reaction; MSM, men who have sex with men.

The management of mpox in immunocompromised patients necessitates tailored treatment strategies. Future research should prioritize the development of evidence-based guidelines for managing severe and atypical cases, particularly in patients with underlying immunosuppression. Such guidelines should address early surgical intervention, optimal wound care, and the role of antiviral or adjunctive therapies to reduce morbidity and improve long-term outcomes.
